# On the In-Growing Toe-Nail

**Published:** 1855-08

**Authors:** G. M. Humphry

**Affiliations:** Cambridge


### *On the In-Growing Toe-Nail.*

[To the Editor of the Medical Times and Gazette.]

Sir,—I have, in the last year or two, read so many letters in the *Medical Times*, and other journals, on the subject of "the ingrowing toe-nail," proposing a variety of painful operative procedures, such as removal of part of the nail, cutting off the everhanging edge of skin, cauterizing the fungous growth, as it is called, etc.; and I have seen so many patients who have suffered under this troublesome complaint for months, and even years, in spite of their having been subjected to much torture, under the hands of good, even most eminent Surgeons, that I think it right to bring before your readers a simple and effectual remedy. It is nothing more than a piece of silver, rolled out sufficiently thin to admit of being bent to the required shape, yet sufficiently firm to bear moderate pressure. This should be nearly the length of the nail, from a quarter to half-an-inch wide, and bent into somewhat of an S shape, or rather thus, 

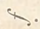

. The lower end (*b*) is, with the aid of a pair of forceps, to be carried down between the overhanging ulcerated skin and the nail, and hooked under the rough edge of the latter. The upper end (*a*) is then carried outwards, and secured in that position by a strip of plaster, and a bandage round the toe. By this means, the inverted edge of the nail and the skin are effectually kept from one another, and pressed in opposite directions. The nail, is a little elevated, and the "fungous growth" very soon shrinks under the pressure of the metal, and assumes a healing aspect. Often, when the silver is well adjusted, the patient is able to walk about with comparative ease immediately afterwards. I do not interfere with it for several days, when a marked improvement is usually found to have taken place. The silver is readjusted with greater ease, and allowed to remain a longer time. Gradually the ulcer heals, and the nail grows up in more natural shape and appearance. It is well, however, to continue the use of the silver for some time; and, after the sore has quite healed, it is well to insert a piece of lint, or a small flat piece of silver, under the edge of the nail, to prevent the tender cicatrix being fretted by it, and to keep down the skin. The patient should be directed to avoid tight shoes, and not to cut the corner of the nail low down. In some bad cases, it has been necessary to keep the patient quiet, or in bed, for a short time; and, in a few, to prepare the way for the silver by the introduction of a piece of lint, secured by a strip of plaster.

There may be nothing novel in the plan here recommended, but it certainly is not known and adopted so generally as it deserves to be. It suggested itself to me when reflecting upon the nature and causes of the complaint, and endeavoring to find some better means of treatment for this very painful and annoying malady than those I had seen employed, feeling certain that the removal of the side of the nail, or any portion of it, which is usually done, must be wrong, for the simple reason that the skin soon occupies the vacancy so caused, and is most likely to be again fretted by the nail growing up into its former place. Since I adopted the above plan, some years age, I have found little difficulty in the treatment of these cases, have instructed some patients to carry our their own cure, and have not failed in a single instance. The size and exact shape of the piece of silver must, of course, be regulated according to the case, and a little nicety of manipulation is required to insinuate it between the ulcerating skin and the nail, and hook it under the edge of the latter, without inflicting much pain in the exquisitely tender state of the part.

I am, etc. G. M. Humphry.

*Cambridge*, June 27, 1855.

